# Endosomal trafficking of the receptor tyrosine kinase MuSK proceeds via clathrin-dependent pathways, Arf6 and actin

**DOI:** 10.1111/febs.12309

**Published:** 2013-05-23

**Authors:** Susan Luiskandl, Barbara Woller, Marlies Schlauf, Johannes A Schmid, Ruth Herbst

**Affiliations:** 1 Center for Brain Research, Medical University of ViennaAustria; 2 Institute of Immunology, Medical University of ViennaAustria; 3 Center for Physiology and Pharmacology Department of Vascular Biology, Medical University of ViennaAustria

**Keywords:** Arf6, clathrin-independent endocytosis, endocytosis, MuSK, receptor tyrosine kinase

## Abstract

Muscle-specific kinase (MuSK), a receptor tyrosine kinase, is the key player during the formation of the neuromuscular junction. Signal transduction events downstream of MuSK activation induce both pre-and postsynaptic differentiation, which, most prominently, includes the clustering of acetylcholine receptors at synaptic sites. More recently, regulated MuSK endocytosis and degradation have been implicated as crucial events for MuSK signalling activity, implicating a cross-talk between signalling and endocytosis. In the present study, we use a live imaging approach to study MuSK endocytosis. We find that MuSK is internalized via a clathrin-, dynamin-dependent pathway. MuSK is transported to Rab7-positive endosomes for degradation and recycled via Rab4-and Rab11-positive vesicles. MuSK activation by Dok7 mildly affects the localization of MuSK on the cell surface but has no effect on the rate of MuSK internalization. Interestingly, MuSK colocalizes with actin and Arf6 at the cell surface and during endosomal trafficking. Disruption of the actin cytoskeleton or the proper function of Arf6 concentrates MuSK in cell protrusions. Moreover, inhibition of Arf6 or cytoskeletal rearrangements impairs acetylcholine receptor clustering and phosphorylation. These results suggest that MuSK uses both classical and nonclassical endosomal pathways that involve a variety of different components of the endosomal machinery.

**Structured digital abstract:**

- MuSK and Arf6 colocalize by fluorescence microscopy (View Interaction: 1, 2)
- MuSK and Rab4 colocalize by fluorescence microscopy (View interaction)
- MuSK and Rab11 colocalize by fluorescence microscopy (View interaction)
- MuSK and Rab7 colocalize by fluorescence microscopy (View interaction)

## Introduction

A neuromuscular junction (NMJ) forms as a result of intercellular interactions between a motor neurone, a muscle fibre and a Schwann cell. Signals from the nerve induce postsynaptic differentiation of the muscle membrane and, in turn, signals from the muscle induce presynaptic nerve terminal differentiation 1, 2.

The muscle specific receptor tyrosine kinase (RTK) MuSK is a key player during NMJ formation. MuSK is essential for NMJ formation because newborn mice lacking *MuSK* do not move or breathe and, consequently, die shortly after birth 3. Levels of acetylcholine receptor (AChR) expression are normal, although AChR clusters or other postsynaptic specializations are missing. In addition, motor axons fail to stop in *MuSK* mutant mice. Axons continue to grow and fail to form arborized nerve terminals. MuSK is activated by the large extracellular matrix protein agrin, which is synthesized by motor neurones, transported in motor axons, released from nerve terminals and deposited in the synaptic basal lamina 4–7. Similar to *MuSK*^*−/−*^ mice, mice lacking *agrin* fail to form NMJs and die at birth as a result of respiratory failure 8. Agrin does not bind MuSK directly but interacts with Lrp4, a member of the low-density lipoprotein receptor family 9, 10. Lrp4 binds MuSK and the interaction of Lrp4 with agrin activates MuSK via an unknown mechanism. *Lrp4* mutant mice also lack all forms of pre-and postsynaptic specializations 11. Taken together, agrin, Lrp4 and MuSK are the key players for NMJ formation and represent the primary scaffold at the developing NMJ that initiates both postsynaptic and presynaptic differentiation.

Current models suggest that MuSK is one of the first proteins present at the site of innervation, providing a primary scaffold for AChR clustering and NMJ formation 12. A signalling cascade requiring MuSK kinase activity and at least one additional tyrosine kinase has been implicated in AChR clustering 13–15. More recently, it was proposed that MuSK endocytosis is required for MuSK signalling 16. Zhu *et al*. 16 showed that agrin induced rapid internalization of MuSK. The expression of dominant-negative dynamin reduced agrin-stimulated MuSK internalization and AChR clustering. These results conform with previous studies of other RTKs, where ligand-induced internalization has been related to signalling events. Retrograde signalling mediated by the neurotrophin receptor TrkA currently represents the best system, supporting the idea that specific signals are generated by endosomes. It was reported that cell surface TrkA promotes nerve growth factor-induced survival, whereas internalized receptors are more active in the promotion of differentiation 17. Similarly, ligand-mediated endocytosis of the epidermal growth factor (EGF) receptor was shown to be required for EGF-induced signalling 18, 19. Current hypotheses propose that membrane trafficking controls signal transduction in one of two ways: (a) it controls the magnitude of the response or (b) it controls the specificity of the response.

In the present study, we aimed to examine the molecular mechanisms involved in MuSK endocytosis and its role during MuSK-dependent AChR clustering. Using an approach that specifically labels MuSK surface receptors, we showed that ligand-independent MuSK internalization preferentially occurs via a clathrin-dependent pathway. Dok7-dependent activation of MuSK does not alter the rate of internalization but favours a localization of MuSK in caveolin-specific structures. We observed a colocalization of MuSK with the small GTPase Arf6 on the cell surface and during endocytosis. Inhibition of the action of Arf6 leads to an accumulation of MuSK in cell protrusions and interferes with agrin-induced AChR clustering, supporting the notion that Arf6 plays an important role during MuSK endocytosis and function.

## Results

### *In vivo* imaging of MuSK endocytosis

**Fig 1:**
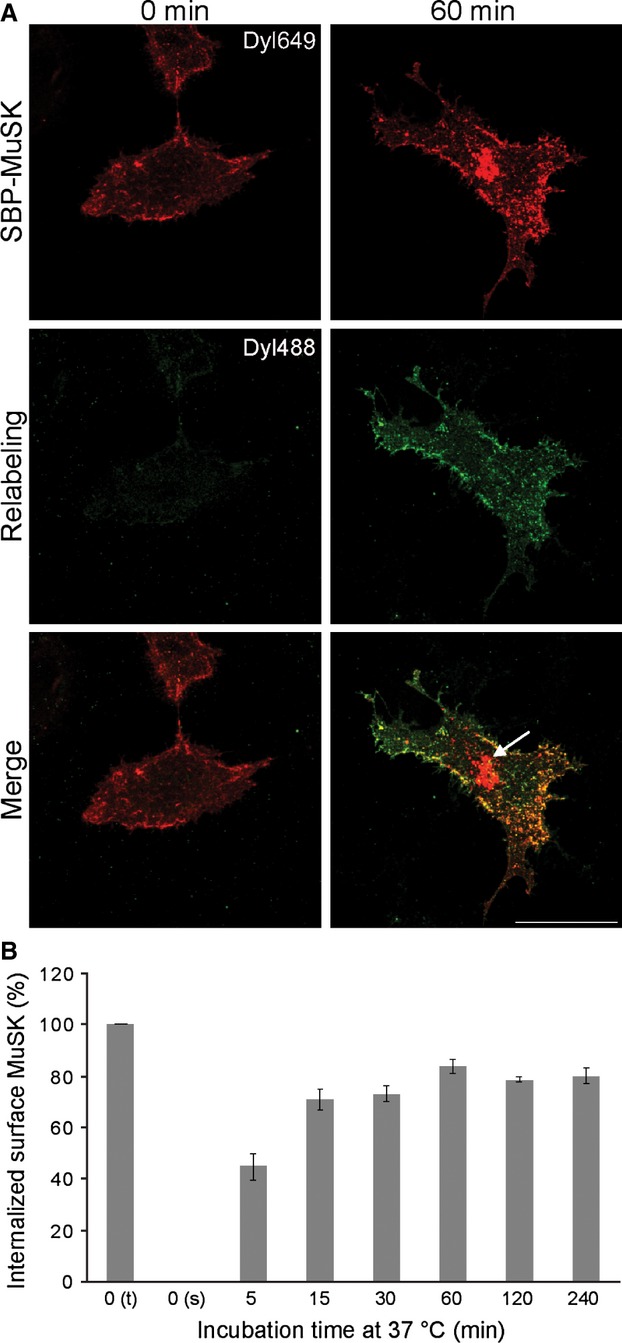
Visualization of SBP-MuSK endocytosis in a heterologous cell system. (A) COS-7 cells were transiently transfected with SBP-MuSK and cells were stained at 4 °C with streptavidin-conjugated to DyLight 649 (red) followed by incubation at 37 °C for different time points, which allows endocytosis to occur. Newly-synthesized surface MuSK was relabelled with streptavidin-conjugated to DyLight 488 (green). (B) To quantify MuSK endocytosis, transfected COS-7 cells were stained with streptavidin-conjugated to DyLight 649 followed by incubation for different time periods at 37 °C. The remaining surface staining was stripped off. Cells were harvested and internalized MuSK was detected by FACS. A quantification of the detected MuSK signal is shown. t, total surface; s, surface after stripping. Error bars indicate the SEM (*n* = 4).

To study MuSK endocytosis, we decided to use an approach where we could specifically label surface receptors and follow their route of internalization by fluorescence microscopy 20. Accordingly, we inserted a streptavidin-binding sequence (SBP), called SBP-Tag, into the extracellular domain of MuSK. The SBP-MuSK construct was expressed in *MuSK^*−*/*−*^* muscle cells to confirm its ability to induce downstream signalling. Figure S1A,B shows that agrin-treatment induced MuSK activation and, consequently, AChR phosphorylation and clustering. To perform an initial dissection of the MuSK endocytosis pathway, we used a simple heterologous system (i.e. COS-7 cells). SBP-MuSK was expressed in COS-7 cells and surface receptors were labelled at 4 °C with DyLight 649-conjugated streptavidin. Cells were incubated for different time periods at 37 °C, at which temperature endocytosis occurs. Newly-synthesized MuSK was stained with DyLight 488-conjugated streptavidin and the labelled receptors were subsequently imaged. As shown in Fig. 1A, labelled SBP-MuSK was restricted to the cell surface without incubation at 37 °C. Increasing incubation periods at 37 °C lead to an accumulation of MuSK in vesicular structures, especially in the perinuclear region, resulting in a pronounced decrease in labelled surface MuSK (Figs 1A and S1C). To quantify MuSK endocytosis over time, we labelled surface MuSK and endocytosis was allowed to proceed at 37 °C for different time periods. Subsequently, streptavidin was stripped off the remaining MuSK surface molecules and intracellular SBP-MuSK was quantified by fluorescence-activated cell sorting (FACS). Figure 1B shows that 40% of surface MuSK was internalized within 5 min and that internalization continuously increased for the first 60 min, reaching a plateau at that time point. To validate that COS-7 cells are suitable for studying MuSK endocytosis, we performed biotinylation experiments to determine MuSK turnover. We found that MuSK turnover is similar in COS-7 cells transiently expressing MuSK compared to C2 muscle cells expressing endogenous MuSK (Fig. S1D,E).

RTK endocytosis is regulated by ligand-induced activation, which usually leads to increased internalization and sorting into specific endosomal pathways 21. Because MuSK cannot be activated by agrin in non-muscle cells 7, we used dominant-negative and constitutive-active mutants of MuSK to determine the effect of MuSK activation on MuSK internalization. Unexpectedly, the activation status of MuSK did not affect the rate of internalization in COS-7 cells (Fig. S1F,G). This observation was supported by biotinylation experiments, which revealed a similar turnover of MuSK wild-type and kinase mutants (Fig. S1D). These results demonstrate that MuSK endocytosis can be studied in living COS-7 cells and that turnover and internalization is similar to that in muscle cells and independent of its activation status.

### MuSK internalization is clathrin-and dynamin-dependent

**Fig 2:**
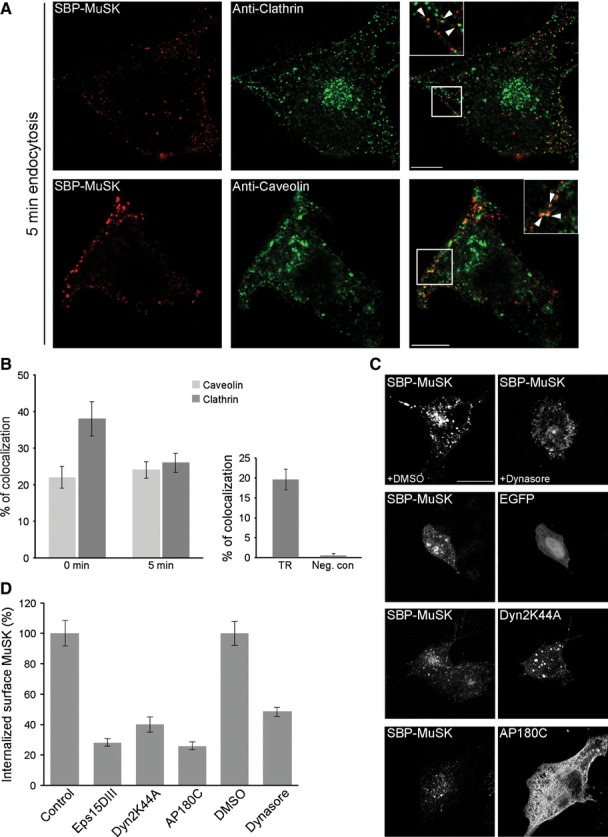
MuSK internalization proceeds via a clathrin-dependent pathway. (A) To determine whether surface MuSK internalizes via clathrin-or caveolin-positive routes, COS-7 cells were transiently transfected with SBP-MuSK. Surface MuSK was stained with Cy3-conjugated streptavidin (red) at 4 °C followed by incubation at 37 °C for 5 min. Endogenous clathrin and caveolin were visualized by antibody staining. MuSK partially colocalizes with these markers (arrowheads in insets). Scale bar = 25 μm. (B) Quantification of MuSK/clathrin, MuSK/caveolin and MuSK/transferrin colocalization using a threshold-and object-based colocalization analysis (as described in the Materials and methods). Colocalization of MuSK with clathrin and caveolin was analyzed at 0 and 5 min of endocytosis. Transferrin and MuSK colocalization was analyzed at 5 min of endocytosis. The peroxisomal marker PTS2-GFP was used as a negative control. Error bars indicate the SEM (*n* ≥ 19; from at least two independent experiments). (C) To determine whether dynamin or clathrin are involved in MuSK internalization, COS-7 cells were either treated with the dynamin specific blocker dynasore or co-transfected with GFP-tagged Dyn2K44A or Myc-tagged AP180C. Surface MuSK was stained with streptavidin conjugated-DyLight 649 (red) at 4 °C followed by incubation at 37 °C for 30 min and subsequent stripping of the remaining surface MuSK molecules. Scale bar = 25 μm. (D) To quantify the blockage of MuSK internalization, COS-7 cells were transiently transfected with SBP-MuSK alone or, as indicated, together with AP180C, Eps15DIII or Dyn2K44A. Cells treated with dimethylsulfoxide (DMSO) or dynasore, were stained with streptavidin-conjugated DyLight 649 followed by incubation at 37 °C for 5 min. The remaining surface staining was stripped off and cells were harvested for intracellular fluorescence detection by FACS. A quantification of internalized MuSK is shown. The control sample denotes GFP-positive cells presenting a streptavidin signal. The dimethylsulfoxide sample represents streptavidin-positive cells treated with the solvent dimethylsulfoxide only. Error bars indicate the SEM (*n* ≥ 5).

A classical pathway of endocytosis occurs via clathrin-coated pits. This endocytic pathway is responsible for the internalization of nutrients, pathogens, growth factors and receptors 22. In particular, ligand-stimulated endocytosis of RTKs is often dependent on clathrin. To test whether MuSK endocytosis occurs via clathrin-coated pits, we determined the colocalization between SBP-MuSK and classical markers of the clathrin-dependent pathway, such as clathrin and transferrin receptor (Figs 2A and S2A). SBP-MuSK was labelled and internalization was imaged after 5 min. We observed a distinct colocalization of MuSK with these marker proteins. To extend these studies, we performed similar experiments with marker proteins of the nonclassical, clathrin-independent, lipid raft-dependent route, such as caveolin, cholera toxin B and CD59 (Figs 2A and S2A). We detected a similar colocalization of MuSK using these markers. A quantification using a threshold-and object-based colocalization analysis (as described in the Materials and methods) revealed a strong colocalization of MuSK with clathrin and a weaker colocalization with caveolin at the cell surface. Upon internalization, the colocalization between MuSK and clathrin is reduced but remains unchanged for MuSK and caveolin (Fig. 2B). A similar colocalization is observed between MuSK and the clathrin-specific marker transferrin. We further analyzed and quantified MuSK endocytosis by confocal microscopy, as well as by FACS as described above. Accordingly, we co-expressed SBP-MuSK with dominant-negative constructs or treated SBP-MuSK expressing cells with inhibitory compounds (Fig. 2C,D). Transferrin was used as a positive control (Fig. S2B). The expression of the carboxy-terminal domain of AP180 (AP180C), which inhibits clathrin-dependent endocytosis by sequestering clathrin, greatly reduced MuSK endocytosis. Similar effects were observed when expressing dominant-negative Eps15 (Eps15DIII), which is an adaptor protein involved in the regulation of clathrin-dependent endocytosis (Fig. 2D). The large GTPase dynamin 2 is an important regulatory protein within different endocytic pathways. We either treated SBP-MuSK expressing cells with the inhibitor dynasore, which inhibits specifically the GTPase activity of dynamin, or co-expressed the dominant-negative dynamin 2 mutant Dyn2K44A and SBP-MuSK. Blocking the function of dynamin impaired MuSK endocytosis to a similar degree as blocking clathrin. Taken together, MuSK is predominantly internalized via pathways that involve clathrin and dynamin. At the cell surface, MuSK preferentially colocalizes with clathrin, which appears to deliver MuSK rapidly into intracellular compartments.

### MuSK is targeted for degradation via Rab7-positive compartments and recycled via Rab4-/Rab11-positive endosomes

**Fig 3:**
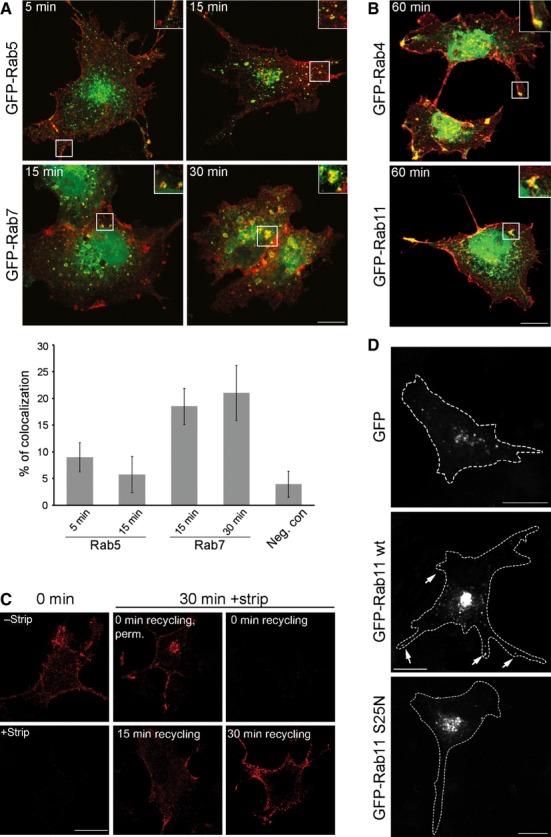
MuSK is transported in Rab7-and Rab4-/11-positive endosomes. (A) To determine whether classical endosomal markers are involved in MuSK trafficking, COS-7 cells were co-transfected with SBP-MuSK together with either GFP-tagged Rab5 (early endosomal compartments, EEA1 positive) or with GFP-tagged Rab7 (late endosomal pathway). Surface MuSK were labelled with DyLight 649-conjugated streptavidin (red) at 4 °C followed by incubation at 37 °C for different time periods. Lower panel: quantification of the colocalization of MuSK with Rab5 or Rab7 after different time points using a threshold-and object-based colocalization analysis (as described in the Materials and methods). MuSK/Arf1 was used as a negative control (neg. con). Error bars indicate the SEM (*n* ≥ 8). (B) COS-7 cells were co-transfected with SBP-MuSK together with either GFP-tagged Rab4 (early recycling) or with GFP-tagged Rab11 (late recycling). Surface MuSK were labelled with DyLight 649-conjugated streptavidin (red) at 4 °C followed by incubation at 37 °C for 60 min. Magnified insets demonstrate a colocalization between MuSK and Rab4 or Rab11. (C) COS-7 cells were transiently transfected with HA-MuSK, stained with an antibody against the extracellular HA-tag followed by incubation at 37 °C for 30 min. The remaining surface antibody staining was stripped off and cells were reincubated at 37 °C for 15 and 30 min (recycling). Cells were fixed and stained with secondary antibodies. Recycled MuSK is detectable at the cell membrane after 15 min. Stripping efficiently removed bound antibodies because no surface MuSK was detectable after 0 min of recycling. Perm, permeabilized. (D) COS-7 cells were co-transfected with either pEGFP, GFP-tagged Rab11 wt or GFP-tagged Rab11 S25N. Surface MuSK was labelled with Cy3-conjugated streptavidin at 4 °C followed by incubation at 37 °C for 120 min. The expression of Rab11 wt increases the recycling of MuSK to the plasma membrane (arrows). Magnified structures are shown as insets. Scale bars = 25 μm.

To analyze the endosomal routing of MuSK, we co-expressed SBP-MuSK and different endosomal marker proteins (Fig. 3). We found a rather low colocalization of MuSK and Rab5 during early endocytosis (after 5 min), as well as at later time points (after 15 min), with a colocalization in the range of 6–9% (Fig. 3A). A similar localization pattern was observed for MuSK and the early endosomal marker EEA1 (data not shown), indicating that MuSK is not transported via classical endosomes or just partially localized to these compartments. However, we found a much higher colocalization of MuSK with the late endosomal marker Rab7, indicating that it is transported via Rab7-positive endosomes to lysosomes similar to other surface receptors (Fig. 3A). Surface molecules are often recycled via Rab4-and/or Rab11-positive endosomes. In support of MuSK recycling, we found that MuSK colocalized with Rab4 or Rab11 (Fig. 3B). To sustain these findings, we expressed MuSK with an extracellular haemagglutinin (HA)-Tag in COS-7 cells. Surface MuSK was labelled by antibody feeding in living cells and endocytosis was allowed to proceed upon transfer to 37 °C. After 30 min, the remaining surface antibody-labelled MuSK was stripped and cells were put back at 37 °C. Recycled MuSK was detected by staining fixed cells with a fluorescently-labelled secondary antibody against the bound HA antibody. As shown in Fig. 3C, after 15 and 30 min, recycled MuSK was detected on the plasma membrane. Recently, it was reported that MuSK recruitment to Rab11-positive endosomes initiates a signalling cascade important for AChR pre-patterning in zebrafish 23. Disruption of the function of Rab11 interferes with MuSK trafficking to endosomes and subsequent MuSK signalling. We therefore tested whether similar effects on MuSK localization are observed in our system upon the expression of Rab11 wild-type or dominant-negative, GDP-locked Rab11 S25N (Figs. 3D and S3). The expression of Rab11 wild-type induced an accumulation of MuSK in the perinuclear region, where it colocalized with Rab11. In addition, MuSK puncta were increasingly observed in the periphery, indicating a recycling back to the cell surface. The expression of Rab11 S25N also recruited MuSK to the perinuclear region but failed to induce MuSK recycling. Taken together, these observations suggest that internalized MuSK is targeted for degradation in lysosomes via Rab7-positive endosomes and also travels along Rab4-/Rab11-positive endosomes back to the cell surface.

### Arf6 and the cytoskeleton play a role during endosomal trafficking of MuSK

**Fig 4:**
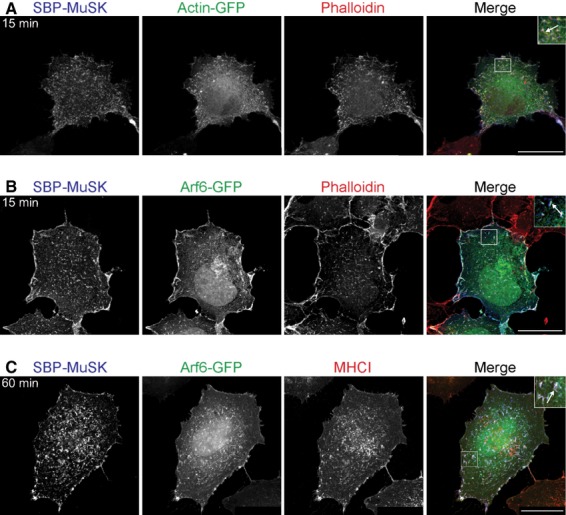
MuSK localizes to structures rich in actin and Arf6. COS-7 cells were transiently transfected with SBP-MuSK. Surface MuSK was labelled with DyLight 649-conjugated streptavidin (blue) at 4 °C followed by incubation at 37 °C for 15 or 60 min. (A) MuSK internalization was visualized in COS-7 cells, which were co-transfected with GFP-tagged actin. The actin cytoskeleton was stained with rhodamine-conjugated phalloidin (red) after cell fixation. (B) MuSK internalization was visualized in COS-7 cells co-transfected with a GFP-tagged Arf6 and stained with phalloidin after cell fixation (red). (C) HeLa cells were transiently transfected with SBP-MuSK together with Arf6-GFP, followed by staining with an antibody against the endogenous Arf6 marker protein MHCI. Magnified structures demonstrating colocalization (arrows) are shown as insets. Scale bar = 25 μm.

In the course of our experiments, we detected a high degree of colocalization between MuSK and actin at the cell surface and during endocytosis, with approximately 30% colocalization after 15 min (Figs 4A and S4A). In particular, we noted a tubular ruffle-like distribution pattern of MuSK and actin. Cytoskeletal rearrangements are crucial for a variety of different endocytic pathways and this is also reflected by characteristic endocytic membrane morphologies. Tubular and vesicular plasma membrane morphologies are found in Arf6-dependent endocytosis 21, 24. Therefore, we asked whether MuSK colocalizes with Arf6 to actin-positive structures. SBP-MuSK and Arf6-GFP or Arf6-HA were co-expressed in COS-7 cells and surface MuSK was labelled (Figs 4B and S4B). Colocalization of MuSK, Arf6 and actin was identified on the cell surface, as well as during MuSK endocytosis, with approximately 40% colocalization of MuSK and actin in the beginning, followed by a gradual decrease over time. MuSK/Arf6 showed 22% colocalization at the start of endocytosis, peaking after 15 min at 27%, followed by a subsequent decrease similar to MuSK/actin colocalization. MuSK was found in Arf6-positive structures after internalization, suggesting that Arf6 is involved in endocytosis rather than recycling. MuSK, Arf6 and actin were colocalized to ruffle-like structures, which did not accumulate perinuclearly. After 60 min of endocytosis, surface and intracellular MuSK was decreased. In the absence of exogenous Arf6, MuSK accumulated in the perinuclear region, whereas the co-expression of Arf6 routed MuSK into ruffles. Interestingly, blocking clathrin-dependent endocytosis by expressing AP180C induced a similar ruffle-like distribution of MuSK. Moreover, MuSK and Arf6 colocalized within these structures (Fig. S4C). Furthermore, we found a colocalization of MuSK, Arf6 and MHCI, which is transported by Arf6-dependent mechanisms (Fig. 4C). Next, we determined whether this colocalization between MuSK and Arf6 is specific by studying the distribution of MuSK relative to Arf1, another member of the Arf family, which has its function in the assembly of cytosolic coat proteins onto Golgi membranes. We found no colocalization between MuSK and Arf1 (Fig. S4D).

**Fig 5:**
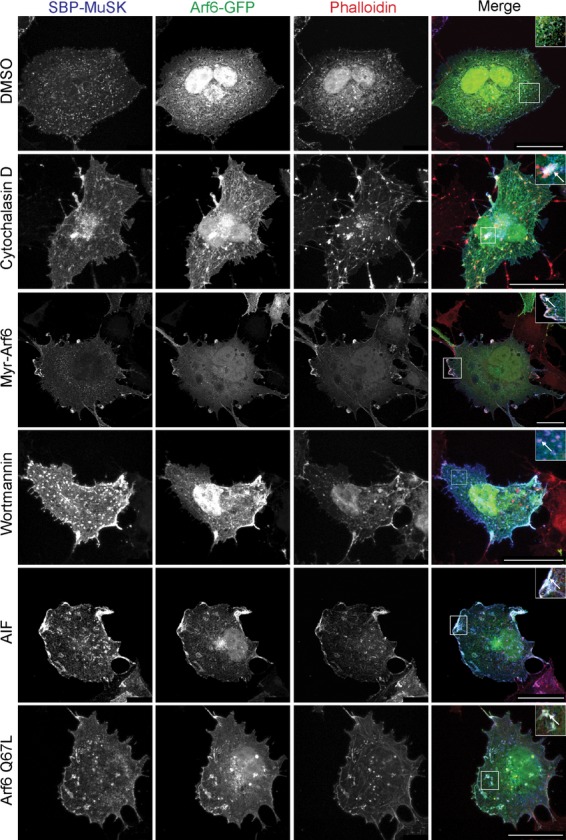
Blocking Arf6 function disrupts MuSK endocytosis. COS-7 cells were transiently transfected with SBP-MuSK and Arf6-GFP. MuSK was labelled with DyLight 649-conjugated streptavidin (blue) at 4 °C followed by incubation at 37 °C for 60 min. After cell fixation, actin was stained with rhodamine-labeled phalloidin (red). Cells were exposed to different treatments: cytochalasin D, myristoylated Arf6 peptide (myr-Arf6), wortmannin, aluminum fluoride (AlF) or co-transfection with the constitutively active mutant Arf6 Q67L. Dimethylsulfoxide treatment was used as a control. MuSK accumulates in cell protrusions close to the cell membrane. Magnified structures demonstrating colocalization (arrows) are shown as insets. Scale bar = 25 μm.

Arf6-dependent endocytosis relies on cytoskeletal rearrangements, on the conversion of phosphatidylinositol (4,5)-bisphosphate (PIP_2_) into phosphatidylinositol (3,4,5)-trisphosphate (PIP_3_), and also on Arf6 activity. Specific inhibitors and activators of these different events were used to study their role during MuSK internalization (Fig. 5). We used cytochalasin D to disturb the actin network and found that MuSK, Arf6 and actin accumulate in large aggregates close to the membrane. These accumulations occurred intracellularly because we did not find a decrease in MuSK internalization (Fig. S5A). When we inhibited Arf6 activation by competition with a synthetic myristoylated Arf6 N-terminal peptide, MuSK aggregated in membrane ruffles together with actin and Arf6 25–27. The conversion of PIP_2_ into PIP_3_ can be blocked using the phosphatidylinositide 3-kinase inhibitor wortmannin. Treatment of SBP-MuSK/Arf6 expressing cells with wortmannin interfered with MuSK internalization and led to an accumulation of MuSK in membranous actin-positive structures. The expression of wild-type Arf6 does not affect the rate of MuSK internalization (Fig. S5B). However, activating Arf6 with aluminum fluoride (AlF) or expressing constitutively active Arf6 Q67L disturbed MuSK internalization. Here, MuSK, Arf6 and actin aggregated in enlarged vacuolar membranes, which resulted from a blocked transport of internalized membranes and cargos to early endosomes. These observations correspond to studies showing that phosphatidylinositide 3-kinase inhibitors such as wortmannin block the transport of MHCI 28. Moreover, studies on Arf6-dependent protein trafficking have shown that the expression of Arf6 Q67L leads to an accumulation of cargo such as MHCI in enlarged positive vacuoles and inhibits their fusion with endosomes 28, 29. In addition, it was previously reported that the G protein activator AlF induces the translocation of over-expressed Arf6 to the cell membrane and stimulates the formation of actin-rich surface protrusions 30, 31. Therefore, the results obtained in the present study suggest that MuSK endocytosis is Arf6-dependent and involves cytoskeletal rearrangements.

### Dok7-induced MuSK endocytosis in heterologous cells

**Fig 6:**
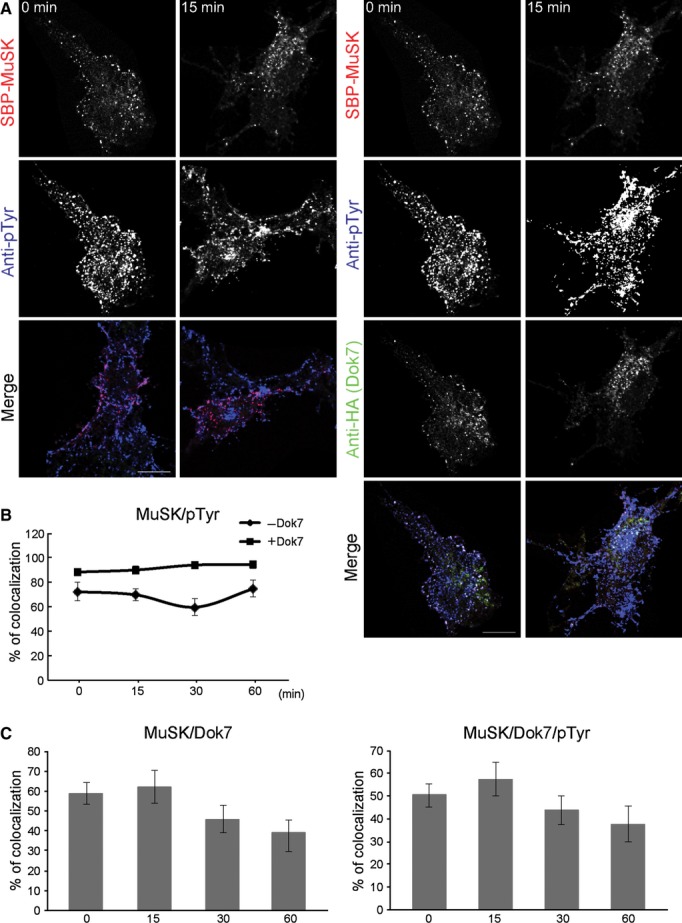
Dok7 expression increases MuSK phosphorylation and its colocalization with phosphotyrosine. (A) MuSK activation by Dok7 was demonstrated by co-expression of SBP-MuSK and HA-tagged Dok7 in COS-7 cells. Surface MuSK was labelled with Cy3-conjugated streptavidin (red) at 4 °C followed by incubation at 37 °C. After cell fixation, the cells were stained with an antibody against phosphotyrosine (pTyr, blue) and an antibody against the HA-tag of Dok7 (green). Scale bar = 25 μm. (B) Quantification of MuSK/pTyr colocalization using a threshold-and object-based colocalization analysis (as described in the Materials and methods) is shown. Error bars indicate the SEM (*n* ≥ 10). (C) Quantification of MuSK/Dok7 and MuSK/Dok7/pTyr colocalization during endocytosis (in min) as in (B). Error bars indicate the SEM (*n* ≥ 10).

**Fig 7:**
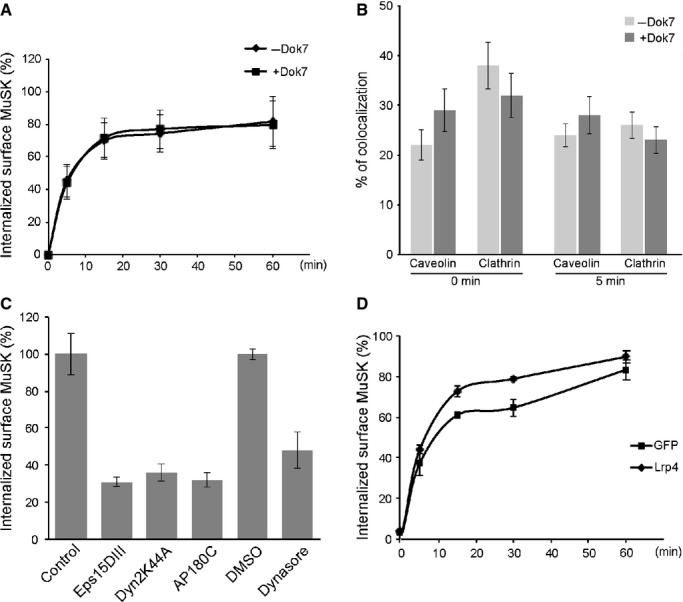
Dok7-dependent MuSK activation does not alter the rate of MuSK internalization but increases the recruitment into caveolin-positive structures on the cell surface. (A) To quantify the influence of Dok7 on the rate of MuSK endocytosis, COS-7 cells were transiently transfected with SBP-MuSK alone or together with Dok7. Surface MuSK was labelled with DyLight 649-conjugated streptavidin at 4 °C followed by incubation at 37 °C for different time periods. The remaining surface staining was stripped off and cells were harvested for intracellular fluorescence detection by FACS. A quantification of internalized MuSK is shown. Error bars indicate the SEM (*n* = 3). (B) To determine whether the presence of Dok7 leads to a switch in the endocytic pathway of MuSK, COS-7 cells were transiently transfected with SBP-MuSK together with Dok7. Surface MuSK was labelled with Cy3-conjugated streptavidin at 4 °C followed by incubation at 37 °C for 5 min. After fixation, cells were stained with an antibody against the endogenous clathrin or caveolin. Quantification of colocalization using a threshold-and object-based colocalization analysis (as described in the Materials and methods) is shown. Error bars indicate the SEM (*n* ≥ 12, from at least two independent experiments). (C) To quantify MuSK endocytosis in the presence or absence of inhibitors, COS-7 cells were transiently co-transfected with SBP-MuSK together with Dok7 and, as indicated, with AP180C, Eps15DIII or Dyn2K44A. Cells, treated with dimethylsulfoxide or dynasore, were stained with streptavidin conjugated-DyLight 649 followed by incubation at 37 °C for 5 min. The remaining surface staining was stripped off and cells were harvested for intracellular fluorescence detection by FACS. Internalized MuSK was quantified. The control sample denotes GFP-positive cells presenting a streptavidin signal. The dimethylsulfoxide sample represents streptavidin-positive cells treated with the solvent dimethylsulfoxide only. Error bars indicate the SEM (*n* = 3). (D) COS-7 cells were transiently transfected with SBP-MuSK and GFP or Lrp4-GFP, respectively. MuSK internalization was detected as described in (A). Error bars indicate the SEM (n = 3).

MuSK is activated by agrin via Lrp4. Lrp4 binds to agrin and, at the same time, interacts with MuSK 9, 10. This presumably results in a structural rearrangement that leads to an autoactivation of MuSK. Furthermore, MuSK activation requires the binding of the cytoplasmic adaptor protein Dok7, which fosters MuSK dimerization and trans-autophosphorylation of the MuSK activation loops 32. This complicated mechanism of activation has made it difficult to study agrin-induced MuSK activation outside of muscle cells. To activate MuSK in COS-7 cells, we co-expressed MuSK and Dok7, which was previously shown to induce MuSK phosphorylation (Fig. S6A) 33. As shown in Fig. 6A,B, tyrosine phosphorylation was greatly increased in the presence of Dok7, as was the colocalization of MuSK and phosphotyrosine. Moreover, we observed a strong colocalization of MuSK and Dok7, as well as MuSK, Dok7 and the phosphotyrosine staining signal, at the cell surface and during subsequent endocytosis (Fig. 6C). Furthermore, we observed a colocalization of MuSK and Arf6, as well as Dok7, after 15 min of internalization (Fig. S6B). Similarly, MuSK, Arf6 and phosphotyrosine colocalized at the cell surface and during internalization (Fig. S6C). Although we detected a strong MuSK activation upon Dok7 expression, the internalization rate of MuSK was not altered (Fig. 7A). After 5 min of internalization, 40% of surface MuSK was detected intracellularly. This increased to approximately 70–80% after 15 and 30 min. To determine whether Dok7-dependent MuSK activation leads to a re-routing of MuSK into a specific endosomal pathway, we examined MuSK colocalization with clathrin and caveolin in the presence and absence of Dok7 (Fig. 7B). We found that surface MuSK localized increasingly towards caveolin-specific compartments in the presence of Dok7 and at the expense of MuSK/clathrin colocalization. Upon MuSK internalization, the MuSK/clathrin colocalization decreased, whereas MuSK/caveolin colocalization remained unchanged. A similar effect on the subcellular localization was observed for a kinase-active MuSK mutant (Fig. S6D). By contrast, Dok7-dependent MuSK activation did not alter the regulation of MuSK internalization (compare Figs 7C and 1B). Interestingly, we found a slightly increased internalization when we co-expressed MuSK and Lrp4, which was also shown to foster MuSK autoactivation 9, 10. Taken together, these results indicate that MuSK activation by Dok7 does not alter MuSK internalization but favours a localization of MuSK in caveolin-positive structures.

### Agrin-induced AChR clustering is dependent on dynamin, Arf6 activity and the cytoskeleton

**Fig 8:**
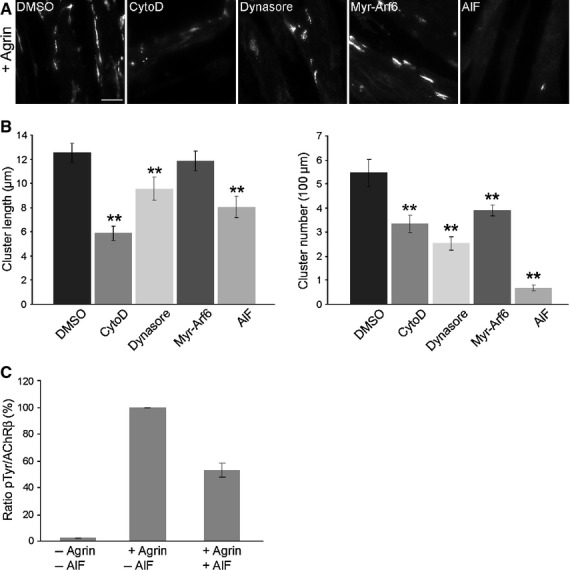
Arf6 modulates agrin-induced AChR clustering. (A) C2 myotubes were stimulated with agrin in the presence of dimethylsulfoxide, cytochalasin D, dynasore, myr-Arf6 or AlF. AChRs were stained with α-BGT and visualized by fluorescence microscopy. Scale bar = 25 μm. (B) Quantification of cluster length and cluster number/100 μm is shown. Error bars indicate the SEM (*n* > 50). (C) C2 myotubes were treated with agrin for 60 min in the absence or presence of AIF. AChRs were purified using an α-BGT pull-down followed by immunoblotting using antibodies against phosphotyrosine (pTyr) and AChR β, respectively. Quantification of AChR β phosphorylation is shown. Error bars indicate the SEM (*n* = 4).

The expression of dominant-negative dynamin blocks agrin-induced MuSK endocytosis and agrin-induced AChR clustering 16. We treated agrin-stimulated C2 myotubes either with the cytoskeletal inhibitor cytochalasin D, the dynamin blocker dynasore, the myristoylated Arf6 peptide to block Arf6 activity, or AIF to activate Arf6, and assayed the resulting AChR clustering (Fig. 8A,B). Inhibition of cytoskeletal rearrangements with cytochalasin D reduced the size and number of AChR clusters by approximately 50% compared to untreated myotubes. Blocking dynamin-dependent endocytosis had a less pronounced effect on the AChR cluster size but reduced the cluster number similar to cytochalasin D. The addition of myristoylated Arf6 peptide had no effect on cluster length but decreased the number of clusters per myotube. By contrast, a myristoylated Arf1 peptide did not affect AChR cluster length or number (Fig. S7). Most severely, the GTPase activating agent AlF, which interferes with membrane fusion during endocytosis via the action of Arf6, almost completely abolished agrin-induced AChR clustering and reduced the length of remaining clusters. In addition, AlF treatment also reduced AChR phosphorylation by approximately 50% (Fig. 8C). These results conform with previous findings implicating the cytoskeleton and dynamin during AChR clustering and now also add Arf6 as an additional regulatory factor.

## Discussion

Activation of MuSK and downstream signalling is a crucial event during AChR clustering at the NMJ. It was recently shown that the regulated removal of surface MuSK by endocytosis influences AChR clustering, implicating a cross-talk between signalling and endocytosis 16. To learn more about the mechanisms of MuSK endocytosis, we have developed an *in vivo* imaging approach to follow MuSK internalization and endocytosis. We demonstrate, in COS-7 cells, that MuSK internalization occurs predominantly via a clathrin-and dynamin-dependent pathway. MuSK accumulates perinuclear in Rab7-positive late endosomes and is also found in Rab4-and Rab11-positive recycling endosomes. Co-expression of MuSK and Dok7 induces MuSK phosphorylation but subsequent endocytosis is not increased. MuSK colocalizes with actin and the small GTPase Arf6 at the cell surface and during endocytosis. Impairment of Arf6-dependent endocytosis using different treatments leads to an accumulation of MuSK in protrusions and interferes with agrin-induced AChR clustering.

### MuSK endocytosis by clathrin-and nonclathrin-dependent pathways

Clathrin-mediated endocytosis (CME) has been considered as the predominant mechanism by which endocytosis of RTKs occurs. More recently, it was demonstrated that ligand-activated RTKs are trapped into lipid rafts and that they are endocytosed via caveolae 22. However, advances in the field have also revealed that additional endocytic pathways exist, although little is known about their exact contribution and importance. Using different treatments to block CME endocytosis, we found that MuSK is predominantly internalized by a clathrin-dependent pathway. Only approximately 30% of MuSK internalizes independent of clathrin. Interestingly, we found that, after increased periods of endocytosis, the internalization of MuSK recovered to approximately 80% in the presence of inhibitory agents (data not shown). This implies that MuSK is able to find alternative routes of entry by switching to different endosomal pathways. Internalization via multiple pathways has been reported for several molecules, such as for the EGF receptor or the TGFβ receptor 22,34–36. It remains unclear which other alternative pathways are used. One of these clathrin-independent endocytosis (CIE) pathways might, in accordance with our observations, include actin and Arf6.

MuSK is colocalized with Arf6 at the plasma membrane, as well as during subsequent endocytosis. Arf6 is a small GTPase that has been implicated as central regulator of CIE 37. This pathway has been studied using the cargo proteins MHCI and CD59 38. MHCI and CD59 both colocalize with Arf6 during early endocytosis and later during recycling 39. The CIE pathway is characterized by the specific lipid composition of the involved endocytic structures. Phosphatidylinositol 4-phosphate 5-kinase, which is activated by Arf6, produces PIP_2_, a critical component of early endocytic compartments 40. A loss of PIP_2_ from these structures is essential for further transport of membrane and cargo to downstream endosomes 41. For that, Arf6 has to be inactivated shortly after internalization. The expression of constitutively active Arf6 Q67L mutant leads to an accumulation of enlarged vacuolar membranes and blocks the progression of endocytosis. Consistent with these observations, the expression of the Arf6 Q67L mutant leads to an accumulation of MuSK in enlarged actin-rich vacuolar membranes. Similarly, other treatments that interfere with Arf6 action, such as wortmannin, which blocks the conversion of PIP_2_ into PIP_3_, or AlF, which activates force-expressed Arf6, have a similar effect. On the other hand, Arf6 has also been implicated in CME as a regulator of coated-pit assembly 42. Therefore, it also appears possible that Arf6 participates in the CME of MuSK. However, when we inhibit CME by the expression of AP180C, MuSK still colocalizes with Arf6 to ruffle-like structures, arguing against a role for Arf6 in the clathrin-dependent endocytosis of MuSK.

During early endocytosis, MuSK not only colocalizes predominantly with clathrin, but also to some degree with caveolin (approximately 20%). After 5 min of endocytosis, the colocalization of MuSK and caveolin remains unchanged, whereas the colocalization of MuSK and clathrin decreases. This can be explained by a trapping of MuSK in lipid rafts or by a slower internalization rate via caveolin-positive compartments. At later time points, MuSK is increasingly found in vesicles containing Rab5 and EEA1. These vesicles are located in the perinuclear region and might represent sorting endosomes or multivesicular bodies, which carry cargo destined for degradation. Accordingly, we noted a strong colocalization of MuSK and Rab7-positive endosomes during late endocytosis, implying that MuSK is degraded via lysosomes. On the other hand, MuSK is also recycled back to the plasma membrane. This transport appears to occur via Rab4-and Rab11-positive endosomes. Blocking Rab11 impairs MuSK recycling and concentrates MuSK in large perinuclear vesicles. This observation contradicts the findings observed in zebrafish, where the inactivation of Rab11 results in a diffuse cytoplasmic localization of MuSK protein. It has been postulated that Wnt11-induced MuSK endocytosis localizes MuSK to Rab11-positive recycling endosomes, thereby inducing a signalling cascade via planar cell polarity components 23. It is currently unclear whether MuSK activation by Wnt11 is Lrp4 dependent and what role Lrp4 plays during AChR pre-patterning. Recent data suggest that Wnt proteins require Lrp4 to activate MuSK 43. This might explain why Rab11 regulates MuSK localization differently in COS-7 cells compared to zebrafish embryos.

### Regulation of MuSK endocytosis by MuSK activity

Similar to other RTKs, MuSK is phosphorylated upon activation, thereby initiating a downstream signalling cascade. However, the mode of activation differs considerably between MuSK and other RTKs, such as EGF receptor, Trk receptors or insulin receptor. First, MuSK is not activated by ligand-binding but rather by the ligand-induced interaction with the co-receptor Lrp4, which presumably results in a conformational rearrangement of MuSK 9, 10. Second, the intracellular adaptor protein Dok7 regulates MuSK activation by supporting MuSK dimerization via its dimeric arrangement, which is facilitated by the Dok7 PH domain 32, 33. It was previously reported that agrin treatment not only stimulates MuSK activation, but also MuSK endocytosis 16. In the present study, we show that MuSK endocytosis occurs independently of its activation status using kinase-inactive and active mutants. Although these results indicate that MuSK activation is not necessary for endocytosis and that a constitutive turnover of MuSK proteins exists, it is also possible that the use of MuSK mutant proteins does not appropriately reproduce agrin-induced MuSK activation. In another approach, we activated MuSK by co-expressing Dok7. Here, we observed a strong tyrosine phosphorylation of MuSK and a co-trafficking of Dok7 and MuSK during early endocytosis. However, the rate of MuSK endocytosis was not affected. On the other hand, Dok7 expression apparently resulted in a preferential association of MuSK with caveolin-specific structures. This is in line with observations showing that activated RTKs are accumulated in lipid rafts before internalization 44, 45. Because we could not detect any effect on the rate of MuSK internalization, it might be possible that Dok7-dependent MuSK activation is not sufficient to alter MuSK endocytosis to the same extent as stimulation with agrin would do (see below). Further studies are required to clarify these results.

### MuSK endocytosis in muscle

It has previously been reported that MuSK endocytosis is induced by agrin-treatment and that this endocytosis depends on dynamin 2 16. We also find a reduction of MuSK endocytosis using either the dominant-negative mutant Dyn2K44A or the chemical blocker dynasore. However, this inhibition is not dependent on MuSK activation because we find the same degree of inhibition when MuSK has been activated by Dok7. This raises the possibility that activation of MuSK with agrin could potentiate the effect of dynamin on MuSK endocytosis. An indication that agrin-Lrp4 might play a role in MuSK endocytosis comes from our data demonstrating an accelerated internalization of MuSK in the presence of Lrp4. Taking the limitations of our model system into account, it will be important to extend our findings to muscle cells using a similar approach of *in vivo* imaging. Such studies will define the endosomal pathways used by MuSK and will help to understand the mechanisms that are involved in MuSK recycling and degradation.

## Materials and methods

### Plasmids

GFP-tagged Arf6 (wt, Q67L) and HA-tagged Arf6 (wt) were kindly provided by Dr J. Donaldson (National Institutes of Health, Bethesda, MD, USA). GFP-tagged Arf1 was a gift from Dr J. Presley (Department of Anatomy and Cell Biology, McGill University, Montreal, Quebec, Canada). GFP-tagged Dyn2a(K44A) and caveolin1 were a gift from Dr A. Helenius (ETH Zurich, Switzerland). GFP-tagged clathrin and Myc-tagged AP180C were a gift from Dr D. Blaas (Max Perutz Laboratories, Vienna, Austria) and GFP-tagged Actin was kindly provided by Dr V. Small (IMBA, Austrian Academy of Sciences, Vienna, Austria). GFP-tagged Eps15(DIII) was a gift from Dr A. Benmerah (Institute Cochin, Université Paris Descartes, Paris, France). GFP-tagged Rab7/Rab11/Rab4 and pLexA-Rab5 were a gift prom Dr M. Zerial (Max Planck Institute of Molecular Cell Biology and Genetics, Dresden, Germany). Rab11 S25N was provided by Dr M. Granato (University of Pennsylvania, Philadelphia, PA, USA). Rab5 was cloned into pEGFP-C2 (Clontech, Palo Alto, CA, USA) using *Eco*RI 46. SBP-MuSK (wt) was generated by introducing the SBP sequence into a 10 amino acid alternative insert in the extracellular domain of MuSK by PCR. pBabe/TK-Dok7-HA and pBabe/Lrp4 9 were a gift from Dr S. Burden (NYU School of Medicine, New York, NY, USA). HA-MuSK was generated by insertion of a HA-Tag at the N-terminus of MuSK after the signal sequence (Asn20). pGFP-TK/Dok7 was generated by insertion of a CMV/EGFP/SV40 cassette and a TK/Dok7/BGHpA fragment into Litmus 29 (New England Biolabs, Beverly, MA, USA). The control plasmid pGFP lacks the TK/Dok7/BGHpA cassette. PTS2-GFP 47 was kindly provided by Dr M. Kunze (Center for Brain Research, Medical University of Vienna, Austria).

### Antibodies and reagents

Antibodies against the extracellular domain (Ig1–2) of MuSK were produced in rabbits (V. Diederichs and R. Herbst, unpublished data). Biotin-and Alexa 594-conjugated α-bungarotoxin (α-BGT) were obtained from Invitrogen (Carlsbad, CA, USA). Rhodamine-conjugated phalloidin, cytochalasin D, dynasore, genistein and wortmannin were purchased from Sigma-Aldrich (St Louis, MO, USA). DyLight 649-conjugated transferrin, Cy3-, DyLight 488-and DyLight 649-conjugated streptavidin were purchased from Jackson ImmunoResearch Laboratories (Ben Harbor, ME, USA). Myristoylated Arf6 N-terminal peptide (2–13) was obtained from Merck (Darmstadt, Germany). Anti-MHCI and anti-CD59 were kindly provided by Dr H. Lassmann (Center for Brain Research, Medical University of Vienna, Austria). Streptavidin agarose beads were obtained from Novagen. Antibodies purchased from commercial sources were: anti-AChR β (Sigma-Aldrich), anti-clathrin (Santa Cruz Biotechnology, Santa Cruz, CA, USA), anti-caveolin (Cell Signaling, Beverly, MA, USA), anti-HA (Sigma-Aldrich), anti-myc 9E10 (Sigma-Aldrich), anti-GFP (Santa Cruz Biotechnology), anti-phosphotyrosine PY-100 (Cell Signaling), anti-phosphotyrosine PY99 (Santa Cruz Biotechnology) and anti-actin (BD Transduction Laboratories, Lexington, KT, USA). Alexa 488-conjugated secondary antibodies were obtained from Invitrogen. Cy3-, DyLight 649-and Texas Red-conjugated secondary antibodies and goat anti-mouse/goat/rat/rabbit horseradish peroxidase-conjugated secondary antibodies were purchased from Jackson ImmunoResearch Laboratories. Soluble neural A4B8 agrin was prepared from HEK 293T as described previously 14.

### Cell culture

COS-7 cells, obtained from ATCC (Wesel, Germany), were cultured in DMEM supplemented with glutamine, 4.5 mg·mL^−1^ glucose, 10% fetal bovine serum and 100 μg·mL^−1^ penicillin/streptomycin. C2 myoblasts were originally obtained from the laboratory of Dr S. Burden (NYU School of Medicine) and have been used in our laboratory since 2002. Cells were grown and differentiated as described previously 48. *MuSK* ^*−/−*^ muscle cells were generated in the laboratory of Dr S. Burden (NYU School of Medicine) and have been used in our laboratory since 2002 14. COS-7 cells were transfected with SBP-tagged MuSK using TurboFect (Fermentas, Glen Burnie, MD, USA). Briefly, cells were plated on coverslips overnight and transfected the next day with 1 μg of DNA and 2 μL of TurboFect in 1 mL of growth medium. Cells were used for experiments 20 h after transfection.

### Endocytosis and recycling assays

To study MuSK endocytosis, transfected cells were starved for 30 min in DMEM, stained with Cy3-, DyLight 649-or DyLight 488-conjugated streptavidin at 4 °C in staining buffer SB (140 mm NaCl, 20 mm Hepes, 1 mm CaCl_2_, 1 mm MgCl_2_ and 5 mm KCl, pH 7.4) containing 1% BSA, followed by incubation at 37 °C for different time points. Cells were fixed with 4% paraformaldehyde (PFA)/NaCl/P_i_ for 10 min at room temperature (RT), mounted in polyvinyl alcohol and visualized using a Leica TCS SP5 spectral confocal microscope with an HCX PL APO CS 63× 1.4 oil objective (Leica Microsystems, Wetzlar, Germany). For the disruption of the actin cytoskeleton, transfected cells were incubated at 37 °C in the presence of 1 μm cytochalasin D. For blocking dynamin function, cells were pre-incubated for 30 min with 80 μm dynasore. After staining at 4 °C, cells were incubated at 37 °C in the presence of dynasore. For disturbing the function of Arf6, cells were pre-treated with 5 μm myr-ARF6 or myr-ARF1 peptide (Merck Millipore, Billerica, MA, USA) for 2 h, with 10 μm wortmannin for 1 h or with AlF (30 mm NaF and 50 μm AlCl_3_) for 30 min. Controls were treated with the solvent dimethylsulfoxide. After staining at 4 °C, cells were incubated at 37 °C in the presence of the peptide, wortmannin or dimethylsulfoxide. To examine MuSK recycling, transfected cells were stained with anti-HA antibodies (dilution 1: 100) in staining buffer SB for 60 min at 4 °C, washed with cold NaCl/P_i_ and incubated with warm SB at 37 °C for 30 min. Cells were placed on ice, stripped with 50 mm glycine, 100 mm NaCl (pH 3.0), washed with cold NaCl/P_i_ and incubated with warm SB at 37 °C for different time periods. Subsequently, cells were rinsed with cold PBS, fixed with 1% PFA/NaCl/P_i_ and stained with Texas Red-conjugated anti-rabbit secondary antibody in SB containing 1% BSA/5% fetal bovine serum. When internalized MuSK was detected, cells were permeabilized with 0.1% Triton/NaCl/P_i_ before antibody staining. Stained cells were mounted in polyvinyl alcohol and visualized using a Leica TCS SP5 spectral confocal microscope with a HCX PL APO CS 63× 1.4 oil objective.

### Immunocytochemistry

Fixed cells were permeabilized with 0.1% Triton/NaCl/P_i_ for 5 min at RT, incubated with blocking solution (NaCl/P_i_ containing 10% fetal bovine serum) followed by incubation with appropriate primary antibodies for 1 h at RT. After washing with NaCl/P_i_, cells were incubated with fluorophore-conjugated secondary antibodies with or without rhodamine-labelled phalloidin.

### Quantification of MuSK endocytosis

COS-7 cells were plated on 12-well plates and transfected with SBP-MuSK and pEGFP (or the appropriate constructs as indicated in the figures) the next day using TurboFect. For Dok7-dependent MuSK activation, cells were transfected with SBP-MuSK and pGFP or pGFP-TK/Dok7. Twenty to 22 h after transfection, cells were starved for 60 min in DMEM. Dynasore (80 mm in dimethylsulfoxide) or dimethylsulfoxide were added at a 1: 1000 dilution during the last 30 min of starvation after washing the respective wells at least four times with DMEM to remove all remaining serum traces. Cells were then stained with DyLight 649-conjugated streptavidin at 4 °C in staining buffer SB (see above) and incubated for the indicated time periods at 37 °C to allow endocytosis. Any remaining surface staining was removed by two sequential washes with NaCl/P_i_ (pH 2.6) for 2 min each. Cells were harvested by incubation with trypsin for 15 min at 4 °C followed by resuspension in ice-cold FACS buffer (NaCl/P_i_ supplemented with 3% fetal bovine serum). Cells were sorted according to their MuSK (DyLight 649) and GFP signals using a BD FACSCalibur flow cytometer (Becton-Dickinson Biosciences, Franklin Lakes, NJ, USA). All experiments included at least duplicate samples and 50 000 cells per sample were counted. Internalized MuSK was calculated as the ratio of double-positive cells (GFP-positive and MuSK-positive) and total GFP-positive cells. The total MuSK signal (without stripping step) was used as a reference value to calculate the percentage uptake for each sample. To show the impact of the different endocytosis inhibitors, the uptake in the control sample (untreated or without dominant-negative construct) was set to 100%.

### AChR clustering assay

C2 myotubes were stimulated with agrin for 8 h and incubated with Alexa 594-conjugated α-BGT to label AChRs for 45 min. Cells were washed with NaCl/P_i_, fixed with 4% PFA, mounted in polyvinyl alcohol and visualized using a Leica DM IRB fluorescence microscope with a 63×/1.4–0.60 oil objective. Cluster length and number was quantified using metamorph imaging software (Molecular Devices, Sunnyvale, CA, USA). To determine the effect of inhibitors on AChR cluster formation, myotubes were stimulated with agrin 4.8 in the presence or absence of inhibitors (cytochalasin D: 1 μm, dynasore: 80 μm, myr-Arf6: 50 μm). For AlF treatment, media was replaced after 4 h with new media containing agrin and fresh NaF and AlCl_3_.

### Pull-down of AChRs

Differentiated C2 cells were starved for 2 h in DMEM followed by incubation with or without AlF (30 mm NaF and 50 μm AlCl_3_) for 30 min. Old medium was removed and new DMEM was added together with fresh AlF and agrin. After 60 min of incubation, cells were lysed with RIPA buffer (1% NP-40, 150 mm NaCl, 50 mm Tris pH 7.5, 0.5% deoxycholate) followed by an AChR pull-down for 1 h with 2 ng·mL^−1^ biotinylated α-BGT followed by incubation with streptavidin agarose beads for 1 h and immunoblotting. The signal intensities were quantified on a FluorS MultiImager (Bio-Rad, Hercules, CA, USA) using Quantity One (Bio-Rad).

### Analysis of colocalization

For each experimental condition, images of more than 10 cells from at least two independent experiments were acquired using a Leica TCS SP5 confocal microscope and were analyzed with imagej (NIH, Bethesda, MD, USA) using a self-generated macro (Doc. S1). In brief, two grey scale images to be analyzed for colocalization were subject to an automatic thresholding based on maximum entropy of the histogram (MaxEntropy threshold method in imagej), followed by area measurement of the thresholded objects (using the ‘Analyze Particles’ routine and a size limit of 5 pixels) for both images. The overlap of the two images was determined by a logical AND operation (using the image calculator routine) and the area of the resulting colocalization objects was again determined using thresholding and the ‘Analyze Particles’ command, as above. The area of these overlapping objects was related to the thresholded area of the relevant marker to calculate a percentage of colocalization. As negative control, we used MuSK/Arf1 colocalization, as well as the co-expression of MuSK and PTS2-GFP (GFP fused to the peroxisomal targeting signal type 2 generating a peroxisomal marker). Because peroxisomes and endosomes do not overlap but exhibit a similar localization pattern, they are perfectly suited to serve as negative control for the colocalization analysis. For all experiments, more than 10 cells were analyzed.

### Statistical analysis

An independent sample Mann–Whitney *U*-test was performed using spss, version 18 (IBM, Armonk, NY, USA) to compare different inhibitory effects on MuSK endocytosis and signalling. *P < 0.05 was considered statistically significant. A double asterisk (**) indicates a significance lower than 0.01%.

## Abbreviations

AChR: acetylcholine receptor
AIF: aluminum fluoride
CIE: clathrin-independent endocytosis
CME: clathrin-mediated endocytosis
EGF: epidermal growth factor
FACS: fluorescence-activated cell sorting
HA: haemagglutinin
MuSK: muscle-specific kinase
NMJ: neuromuscular junction
PIP_2_: phosphatidylinositol (4,5)-bisphosphate
PIP_3_: phosphatidylinositol (3,4,5)-trisphosphate
RTK: receptor tyrosine kinase
RT: room temperature
SBP: streptavidin-binding sequence
α-BGT: α-bungarotoxin

## Supplementary material

**Doc. S1.** Supplementary materials and methods. **Fig. S1.** MuSK turnover in COS-7 cells and C2 myotubes. **Fig. S2.** MuSK colocalization with clathrin and lipid raft markers and transferrin internalization in the presence of clathrin and dynamin inhibitors. **Fig. S3.** Rab11 regulates MuSK recycling. **Fig. S4.** MuSK endocytosis involves the actin cytoskeleton and Arf6. **Fig. S5.** The MuSK internalization is not altered upon cytochalasin D treatment or the expression of Arf6. **Fig. S6.** MuSK activation by Dok7 and its colocalization with pTyr, Arf6 and endosomal markers. **Fig. S7.** Agrin-induced AChR clustering is not affected in Myr-Arf1 treated myotubes.
