# Supplementary material for: Endosomal trafficking of the receptor tyrosine kinase MuSK proceeds via clathrin-dependent pathways, Arf6 and actin
Source: FEBS J. 2013 May 23;280(14):3281–97. doi: 10.1111/febs.12309 (PMC3806275; doi:10.1111/febs.12309)
Supplement: Supplementary file 1 — Doc. S1. Supplementary materials and methods. Fig. S1. MuSK turnover in COS-7 cells and C2 myotubes. Fig. S2. MuSK colocalization with clathrin and lipid raft markers and transferrin internalization in the presence of clathrin and dynamin inhibitors. Fig. S3. Rab11 regulates MuSK recycling. Fig. S4. MuSK endocytosis involves the actin cytoskeleton and Arf6. Fig. S5. The MuSK internalization is not altered upon cytochalasin D treatment or the expression of Arf6. Fig. S6. MuSK activation by Dok7 and its colocalization with pTyr, Arf6 and endosomal markers. Fig. S7. Agrin-induced AChR clustering is not affected in Myr-Arf1 treated myotubes. [file febs0280-3281-sd1.zip › febs12309-sup-0001-FigureS1-S7_docS1.pdf]

the FEBS Journal
WILEY Blackwell
www.febsjournal.org

Endosomal trafficking of the receptor tyrosine kinase MuSK proceeds via clathrin-dependent pathways, Arf6 and actin

Susan Luiskandl, Barbara Woller, Marlies Schlauf, Johannes A. Schmid and Ruth Herbst

DOI: 10.1111/febs.12309

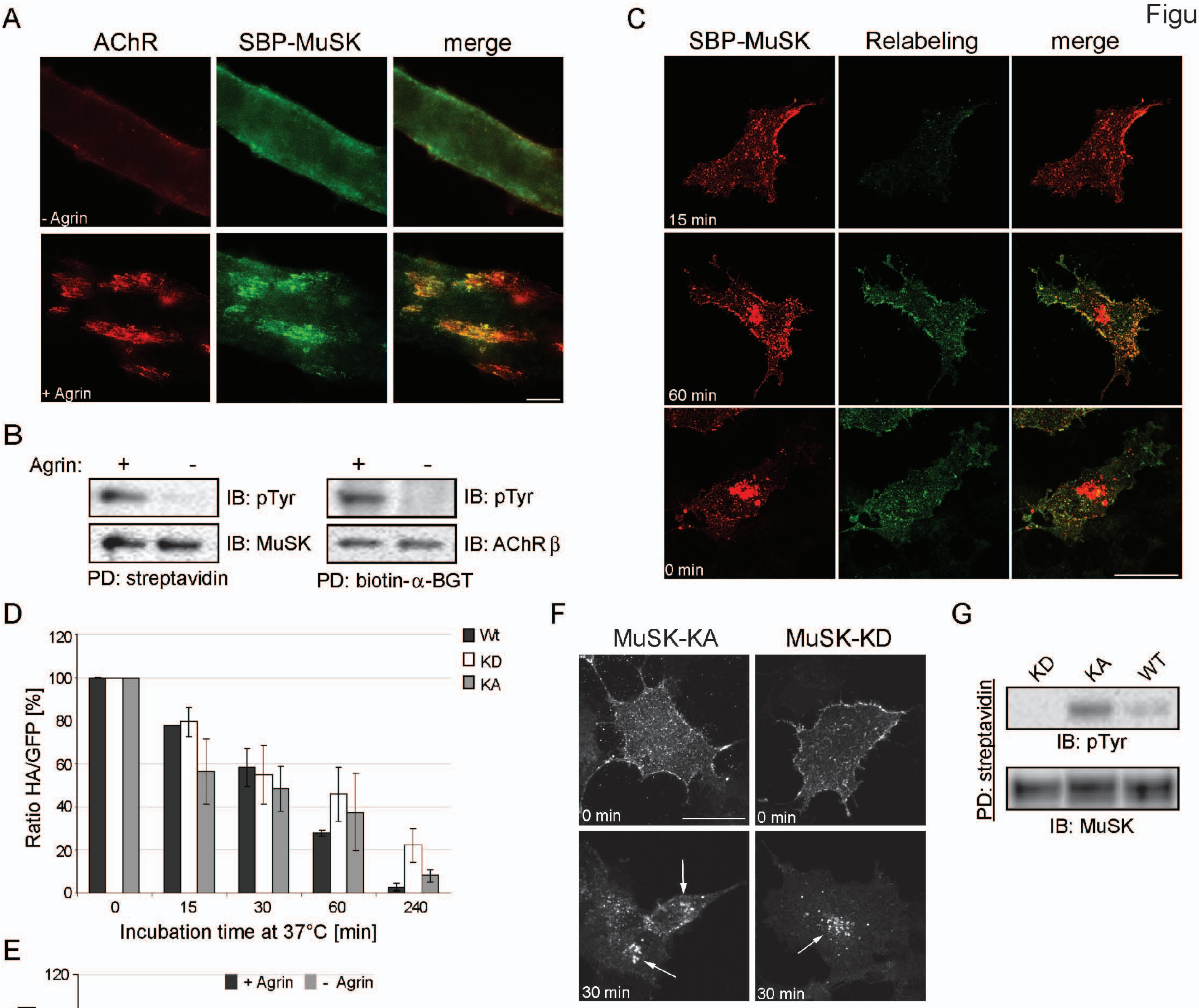

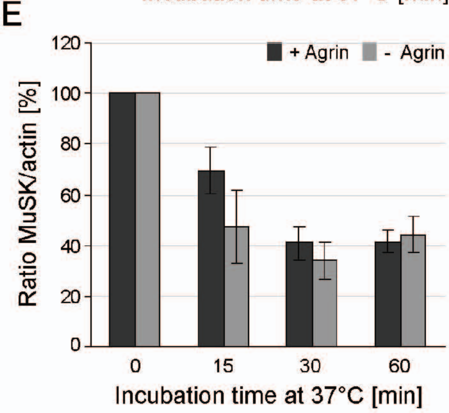

Figure S1: MuSK turnover in COS-7 cells and C2 myotubes. (A) *MuSK*$^{-/-}$ muscle cells expressing SBP-MuSK either unstimulated or stimulated with agrin were stained with DyLight 488-conjugated streptavidin to label MuSK and with Alexa 594-conjugated α-BGT to label AChRs. (B) MuSK and AChRs were isolated from *MuSK*$^{-/-}$ cell lysates expressing SBP-MuSK using streptavidin-agarose or biotin-conjugated α-BGT, respectively. Samples were subjected to SDS-PAGE followed by immunoblotting (IB) with anti-phosphotyrosine (pTyr) antibodies. Blots were reprobed with anti-MuSK or anti-AChR β antibodies. PD, pull-down. (C) To visualize MuSK endocytosis, COS-7 cells were transiently transfected with SBP-MuSK. Cells were stained at 4°C with streptavidin-DyLight 649 (red) followed by incubation at 37°C for different time periods. Newly synthesized surface MuSK was re-labeled with streptavidin-Alexa 488 (green). (D) COS-7 cells were transiently transfected with either wild-type (Wt), kinase-dead (KD, K608A) or kinase-active (KA, LS745/746MT) HA-MuSK constructs, and with pEGFP as transfection control. Surface proteins were biotinylated using Sulfo-NHS-SS-Biotin, streptavidin-precipitated and analyzed by immunoblotting. The ratio of HA/GFP was quantified using Quantity One (Bio-Rad). A quantification of surface MuSK over time is shown. Error bars: SEM (n = 4). (E) To determine the half-life of endogenous surface MuSK in muscle cells, surface proteins were biotin-labeled and C2 myotubes were incubated in the absence or presence of agrin at 37°C for different time points. Labeled proteins were isolated by streptavidin-agarose pull-down and analyzed by immunoblotting with antibodies against MuSK. A quantification of the MuSK/actin ratio is shown. MuSK internalization occurs independent of agrin stimulation. Error bars: SEM (n ≥ 4). (F) To determine whether the kinase activity of MuSK affects its internalization, COS-7 cells were transiently transfected with either wild-type, KD- or KA-SBP-MuSK constructs. Cells were stained at 4°C with streptavidin-DyLight 649 followed by incubation at 37°C for 30 minutes. Both MuSK mutants are internalized independent of their activation status. (G) COS-7 cells transiently expressing either wild-type, KD- or KA-SBP-MuSK were lysed. MuSK was isolated by streptavidin-agarose pull-down (PD) followed by immunoblotting (IB) with antibodies against phosphotyrosine (pTyr) and MuSK. The kinase-active MuSK mutant is strongly phosphorylated compared to wild-type MuSK. Kinase-dead MuSK is not phosphorylated. MuSK mutants -K608A, LS745/746MT- were provided by Dr. Steve Burden. Scale bars, 25 μm.

Figure S2
Luiskandl et al.

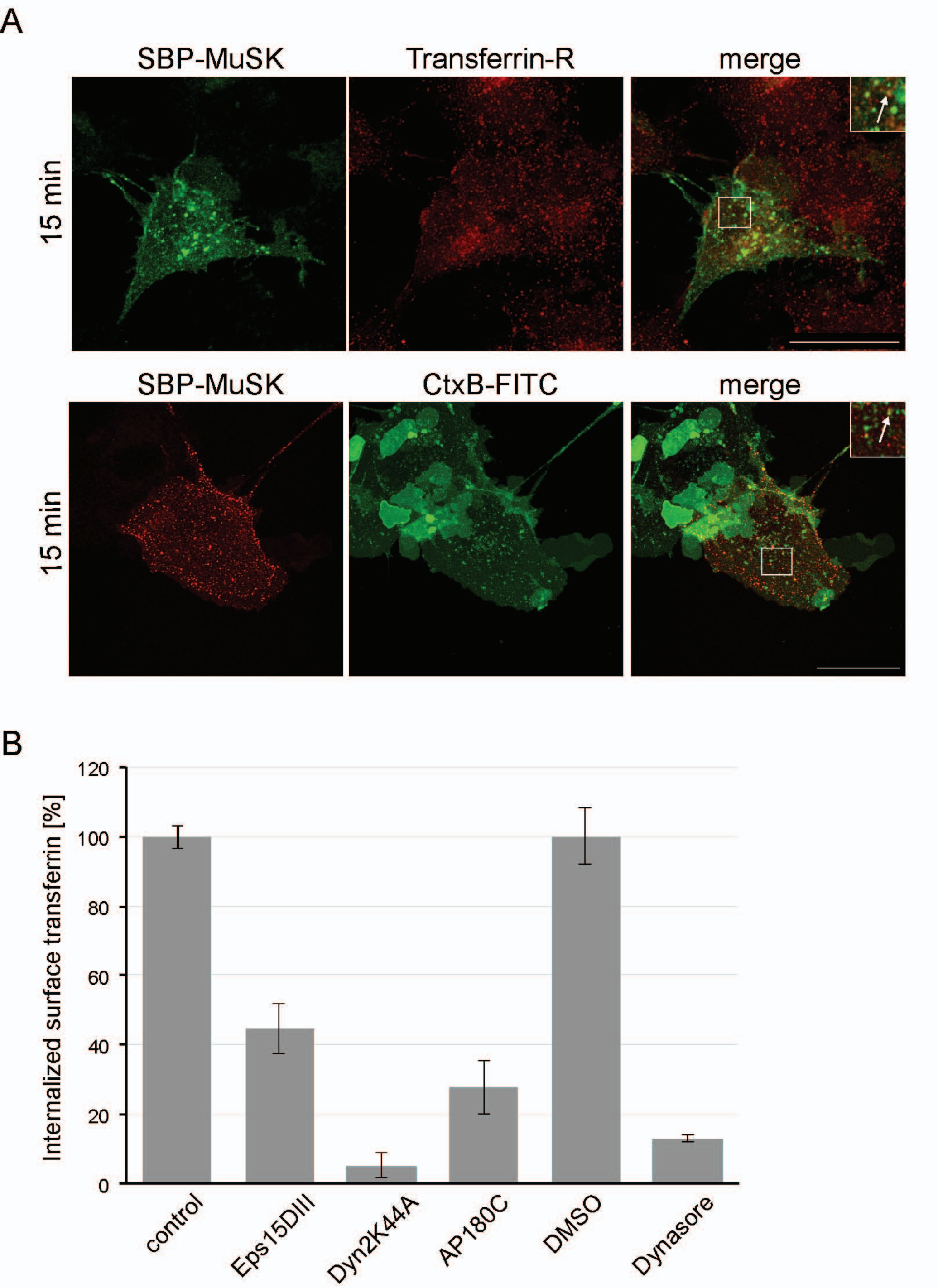

Figure S2: MuSK colocalization with clathrin and lipid raft markers and transferrin internalization in the presence of clathrin and dynamin inhibitors. (A) COS-7 cells were transiently transfected with SBP-MuSK. Cells were stained at 4°C with streptavidin conjugated to DyLight 649 (red) followed by incubation at 37°C for 15 min together with either Rhodamine-conjugated transferrin (Rockland) or with FITC-conjugated CtxB (Sigmal-Aldrich). A weak but distinct colocalization between MuSK and transferrin or CtxB is detected. Magnified structures are shown as insets. Scale bars, 25 µm. (B) COS-7 cells transfected with the indicated constructs or treated with DMSO or dynasore respectively were in vivo labelled with DyLight 649-conjugated transferrin. After 2 min endocytosis cells were stripped and harvested for subsequent FACS analysis. A quantification of internalized transferrin is shown. Error bars: SD ($n \geq 2$).

Figure S3
Luiskandl et al.

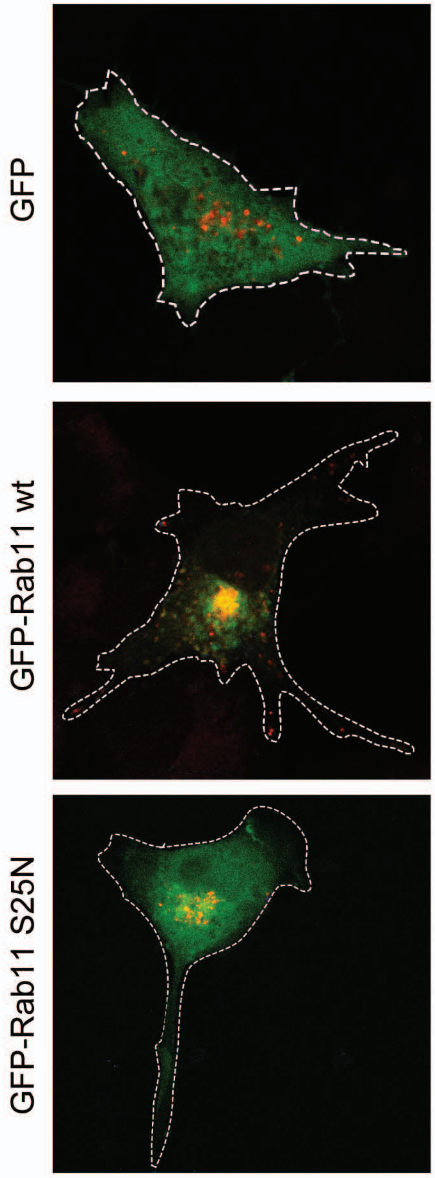

Figure S3: Rab11 regulates MuSK recycling. COS-7 cells were co-transfected with SBP-MuSK and pEGFP or GFP-tagged Rab11 (wt or Rab11 S25N). Surface MuSK was stained with Cy3-conjugated streptavidin at 4°C followed by an incubation at 37°C for 120 minutes.

Figure S4
Luiskandl et al.

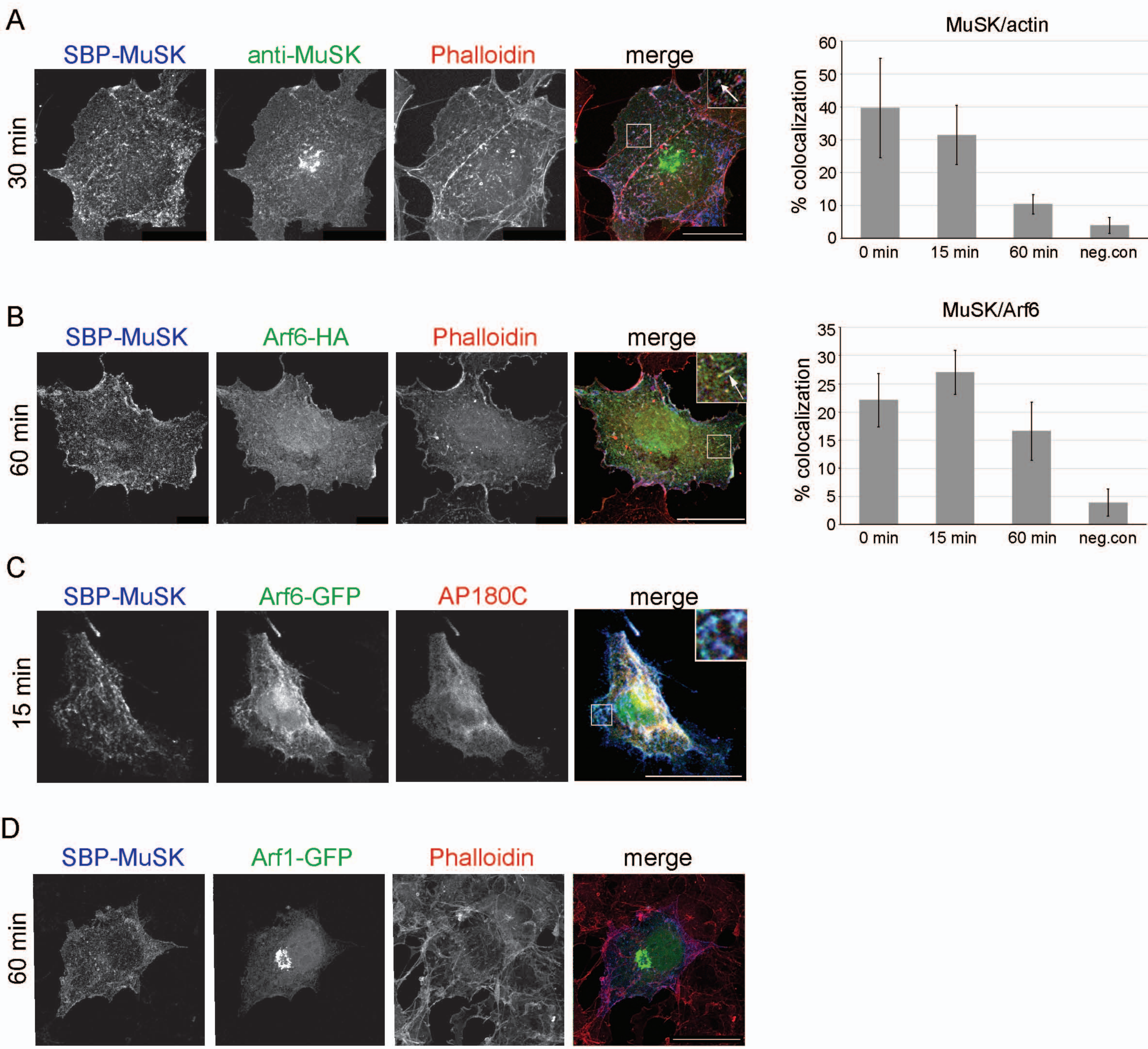

Figure S4: MuSK endocytosis involves the actin cytoskeleton and Arf6. (A) To determine whether the actin cytoskeleton is involved in MuSK endocytosis, COS-7 cells were transiently transfected with SBP-MuSK. Cells were stained at 4°C with DyLight 649-conjugated streptavidin (blue) followed by incubation at 37°C for 30 minutes. After cell fixation cells were stained with Rhodamine-conjugated phalloidin (red) and antibodies against MuSK (green). Threshold- and object-based colocalization analysis as described in Materials and Methods shows a colocalization between MuSK and actin preferentially at the cell surface and during early endocytosis. (B) COS-7 cells were co-transfected with SBP-MuSK and HA-tagged Arf6. After staining of surface MuSK at 4°C (blue) endocytosis proceeded at 37°C for 60 minutes. Cells were fixed and actin was stained with Rhodamine-conjugated phalloidin (red). Threshold- and object-based colocalization analysis as described in Materials and Methods shows a colocalization between MuSK and Arf6 at the cell surface and during endocytosis. (C) Cells were co-transfected with SBP-MuSK (blue), Myc-tagged AP180C (red) and Arf6-GFP (green). Despite AP180C expression MuSK still colocalizes with Arf6 to ruffle-like structures. (D) To confirm that MuSK colocalizes specifically with Arf6, MuSK endocytosis was visualized in cells expressing SBP-MuSK (blue) and Arf1-GFP (green). After fixation actin was stained with Rhodamine-conjugated phalloidin (red). No colocalization between Arf1 and MuSK was observed. Magnified structures are shown as insets. Scale bars, 25 µm. Error bars: SEM.

Figure S5
Luiskandl et al.

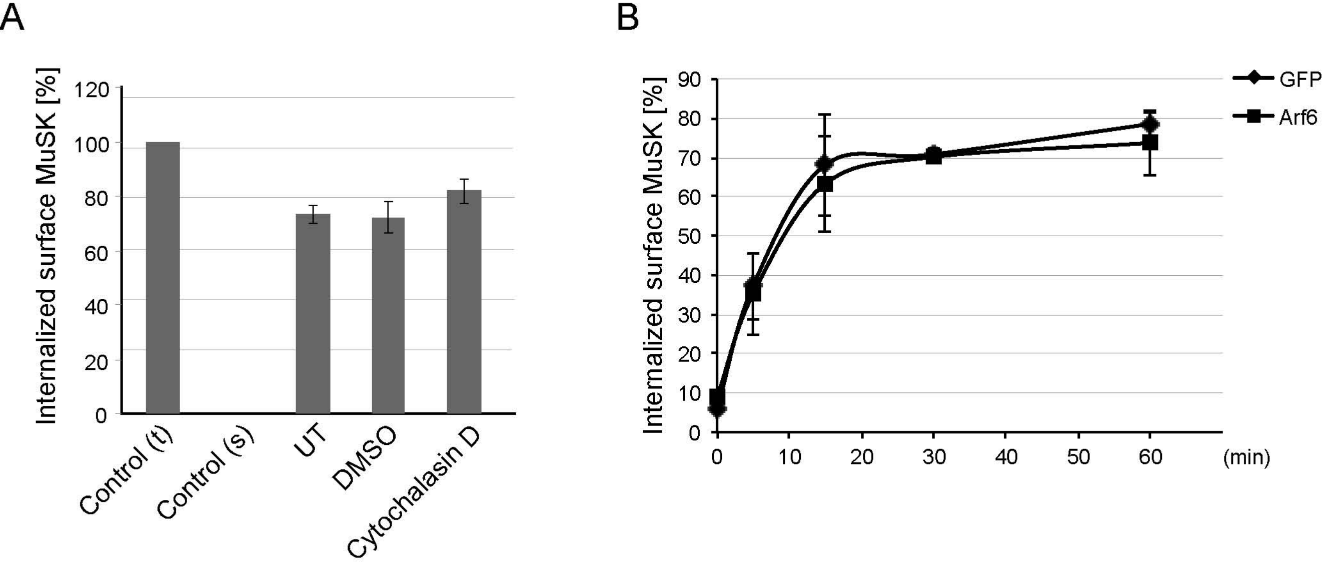

Figure S5: The MuSK internalization is not altered upon cytochalasin D treatment or the expression of Arf6. (A) To quantify MuSK internalization, COS-7 cells were transiently transfected with SBP-MuSK, stained with DyLight 649-conjugated streptavidin followed by an incubation at 37°C for 30 minutes in the absence or presence of inhibitor. Remaining surface staining was stripped off and cells were harvested for intracellular fluorescence detection by FACS. A quantification of internalized MuSK is shown. (t) total surface staining; (s) staining after stripping; UT, untreated. Error bars: SEM (n = 3). (B) COS-7 cells were co-transfected with SBP-MuSK and Arf6-GFP or GFP alone. Cells were stained with DyLight 649-conjugated streptavidin followed by an incubation at 37°C for increasing time periods. Remaining surface staining was stripped off and cells were harvested for intracellular fluorescence detection by FACS. A quantification of internalized MuSK in GFP-positive cells is shown. Error bars: SD (n = 2 in duplicates).

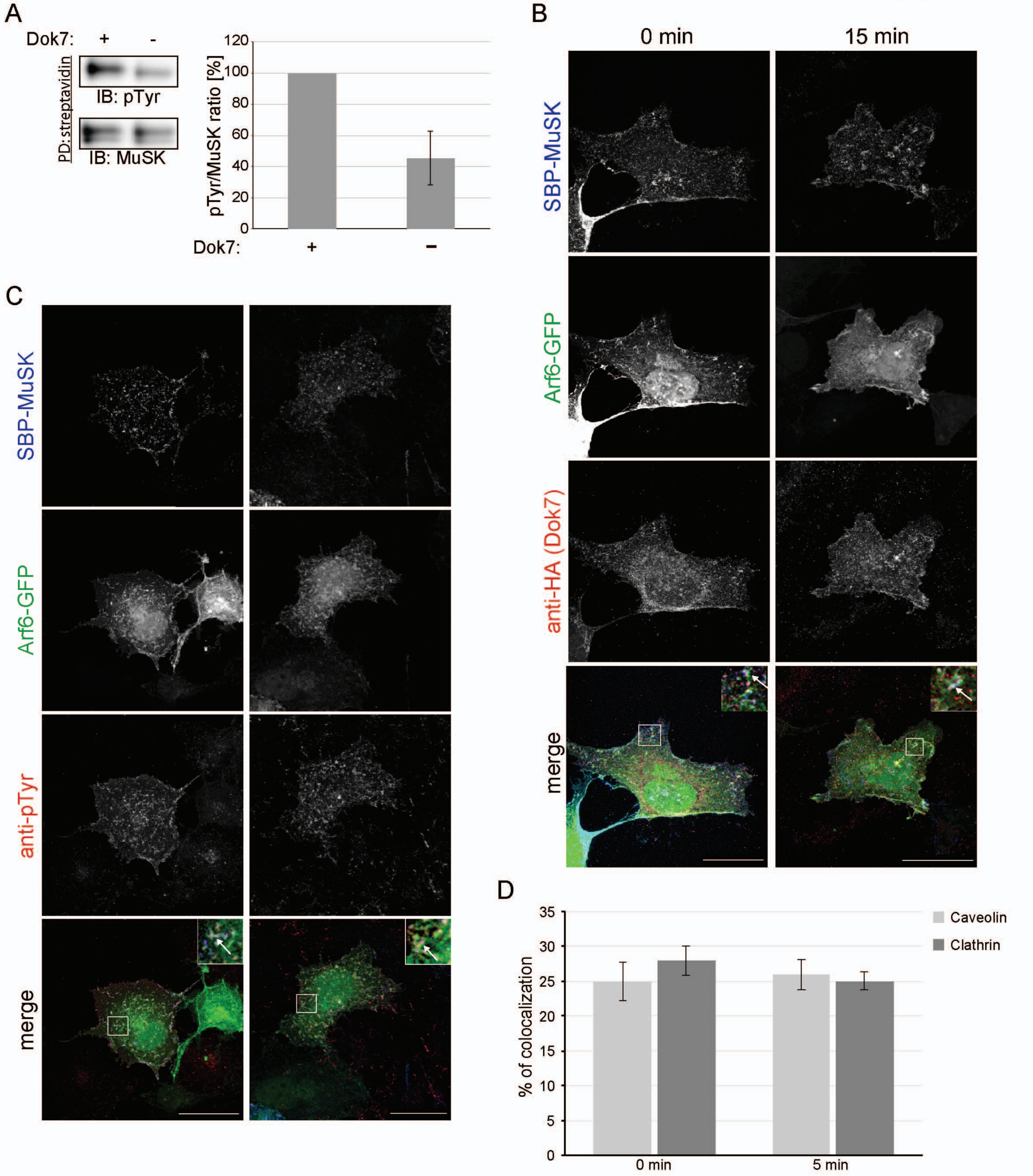

Figure S6: MuSK activation by Dok7 and its colocalization with pTyr, Arf6 and endosomal markers. (A) To determine whether SBP-MuSK is activated by co-expression of Dok7, cells transfected with SBP-MuSK and Dok7-HA were lysed and MuSK was precipitated by streptavidin pull down (PD). Proteins were analyzed by immunoblotting (IB). A quantification of MuSK phosphorylation is shown. Error bars: SEM (n = 3). (B) and (C) To determine the colocalization of MuSK / Arf6 / Dok7 and MuSK / Arf6 / pTyr, cells were co-transfected with SBP-MuSK (blue), Arf6-GFP (green) and HA-tagged Dok7. Surface MuSK was labeled with DyLight 649-conjugated streptavidin at 4°C followed by incubation at 37°C for 15 minutes. After cell fixation, cells were stained with an antibody against HA (red) or phosphotyrosine (pTyr, red). Magnified structures demonstrating colocalization (arrows) are shown as insets. Scale bars, 25 µm. (D) Kinase-active MuSK (LS745,746MT) was expressed in COS-7 cells. Surface MuSK was labeled with CY3-conjugated streptavidin at 4°C followed by incubation at 37°C for 5 minutes. Fixed cells were stained with antibodies against caveolin or clathrin. Quantification of MuSK / clathrin and MuSK / caveolin colocalization was performed using a threshold- and object-based colocalization analysis (as described in Materials and Methods). Error bars: SEM

Figure S7
Luiskandl et al.

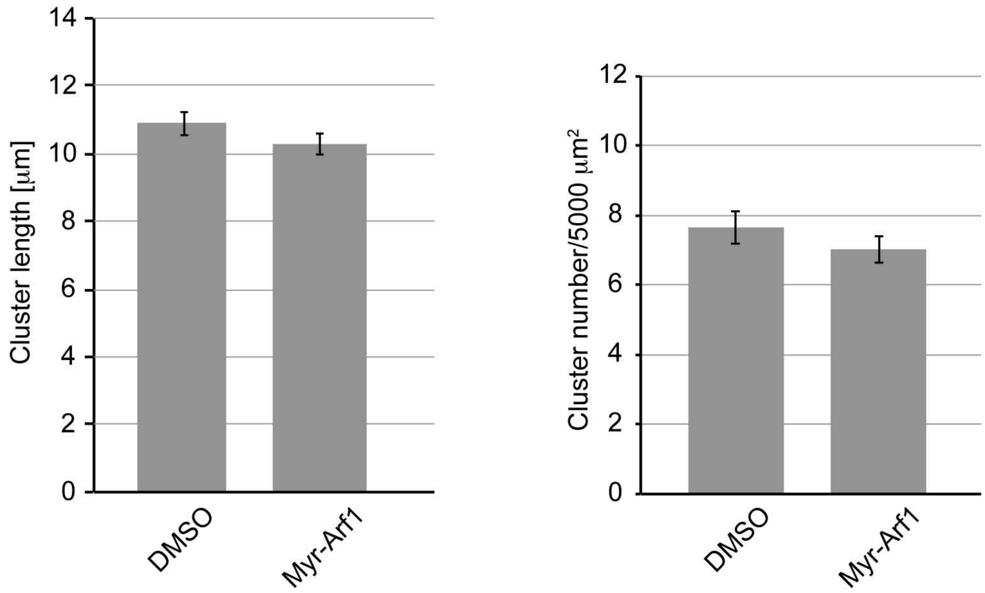

Figure S7: Agrin-induced AChR clustering is not affected in Myr-Arf1 treated myotubes. C2 myotubes were stimulated with agrin in the presence of myr-Arf1. AChRs were stained with alpha-bungarotoxin (α-BGT) and visualized by fluorescence microscopy. Quantification of cluster length and cluster number revealed no difference to untreated myotubes. Error bars: SEM (n > 30).

Doc. S1: Supplementary Methods and Materials

<u>Colocalization Macro for ImageJ:</u>

```
run("Set Measurements...", "area limit redirect=None decimal=3");
run("Set Scale...", "distance=0 known=0 pixel=1 unit=pixel global");
selectImage(1);
setAutoThreshold("MaxEntropy dark");
run("Analyze Particles...", "size=5-Infinity circularity=0.00-1.00 show=Nothing summarize");
run("Convert to Mask");
selectImage(2);
setAutoThreshold("MaxEntropy dark");
run("Analyze Particles...", "size=5-Infinity circularity=0.00-1.00 show=Nothing summarize");
run("Convert to Mask");
imageCalculator("AND create", 1, 2);
rename("colocalization");
setAutoThreshold("MaxEntropy");
run("Analyze Particles...", "size=5-Infinity circularity=0.00-1.00 show=Nothing summarize");
selectWindow("Summary")
```

We established this object-based colocalization analysis, because we had made the observation that intensity-based correlation methods calculating correlation coefficients for pixel intensities in the two channels according to Pearson or modifications thereof, were prone to false positive results or high standard deviations and that an object-based method delivers much better and more robust data. A comparison of different colocalization methods using the ImageJ plugin JACoP clearly revealed the superiority of the object-based method (Bolte & Cordelieres, 2006). However, in this later plugin, only the number of colocalizing objects is evaluated, which gives a high standard deviation for smaller numbers of objects. Therefore, we extended the approach so as to calculate the area of colocalizing objects after a standardized thresholding procedure as percentage of channel 1 or channel 2.

Isolation of surface proteins by biotinylation

The half-life of MuSK was analyzed by using cell-impermeable biotin as described previously (Ehlers, 2000; Yang et al, 2005). Briefly, cells were starved for 30 min in DMEM, surface proteins were biotinylated with 0.5 mg/ml sulfo-NHS-SS-biotin (Pierce) in DMEM at 4°C for 25 min. After washing with cold PBS (supplemented with 1mM $MgCl_2$ and 0.1mM $CaCl_2$, pH 7.9), the cells were incubated at 37°C in DMEM which allows the endocytosis to occur. Cells were washed again and lysed in RIPA buffer. Cell lysates were incubated with streptavidin beads overnight at 4°C. After washing the beads with RIPA buffer, biotinylated proteins were analyzed by immunoblotting.

References

Bolte S & Cordelieres FP (2006) A guided tour into subcellular colocalization analysis in light microscopy. *Journal of Microscopy* **224**, 213-232, doi: 10.1111/j.1365-2818.2006.01706.x.

Ehlers MD. Reinsertion or degradation of AMPA receptors determined by activity-dependent endocytic sorting. Neuron. 2000;28(2):511-25.

Yang XL, Huang YZ, Xiong WC, Mei L. Neuregulin-induced expression of the acetylcholine receptor requires endocytosis of ErbB receptors. Mol Cell Neurosci. 2005;28(2):335-46.
